# Laryngeal mask airway combined with bronchial blocker achieved 1-lung ventilation in a patient with bilateral vocal cord paralysis: A case report

**DOI:** 10.1097/MD.0000000000037409

**Published:** 2024-03-08

**Authors:** Yi Li, Yudong Zhang, Yu Zhang, Lei Meng, Chong Li, Jianli Li

**Affiliations:** a Department of Anesthesiology, Hebei General Hospital, Shijiazhuang, China.

**Keywords:** anesthesia, bilateral vocal cord paralysis, bronchial blocker, laryngeal mask airway, one-lung ventilation

## Abstract

**Introduction:**

One-lung ventilation (OLV) is a commonly used technique to facilitate surgical visualization during thoracic surgical procedures. Double-lumen endotracheal tubes and one-lumen tracheal tube combined with bronchial blocker might lead to intubation-related laryngeal injury.

**Patient concerns:**

In the perioperative period, how to avoid further damage to the vocal cord while achieving OLV during operation is challenging work.

**Diagnosis:**

She was diagnosed with systemic lupus erythematosus, bilateral vocal cord paralysis, and lung tumor.

**Interventions:**

We used a combination of a laryngeal mask airway with bronchial blocker to avoid further damage to the vocal cord when achieving OLV.

**Outcomes:**

At 1-month follow-up, she had fully recovered without obvious abnormalities.

**Conclusion:**

When OLV was required for patients with bilateral vocal cord paralysis, a combination of a laryngeal mask airway with bronchial blocker was considered a better choice.

## 1. Introduction

One-lung ventilation (OLV) is a technique to facilitate optimal surgical conditions during thoracic surgical procedures, which could be achieved by using a double-lumen endotracheal tube (DLT) or endotracheal tube combined with bronchial blocker (BB). Although the DLTs are considered to be the gold standard, DLTs or endotracheal tubes combined with BBs are equivalent efficacy in elective thoracoscopic surgeries.^[1]^ However, both airway devices require the use of a large-bore tracheal tube. As far as we know, the most important factor of intubation-related laryngeal injury is the lumen size of the tracheal tube. Thus, it is a challenge to achieve OLV while avoiding further damage to the vocal cord in patients with bilateral vocal cord paralysis. According to literature reports, laryngeal mask airway (LMA) combined with BB is another method for achieving OLV. In this case, we presented a case with a history of bilateral vocal cord paralysis due to systemic lupus erythematosus (SLE), who was scheduled for lung tumor surgery. We successfully used a combination of an LMA with BB to avoid further damage to the vocal cord while achieving OLV. The reporting of this study conforms to CAse REport guidelines.^[2]^

## 2. Case presentation

**Figure 1.**
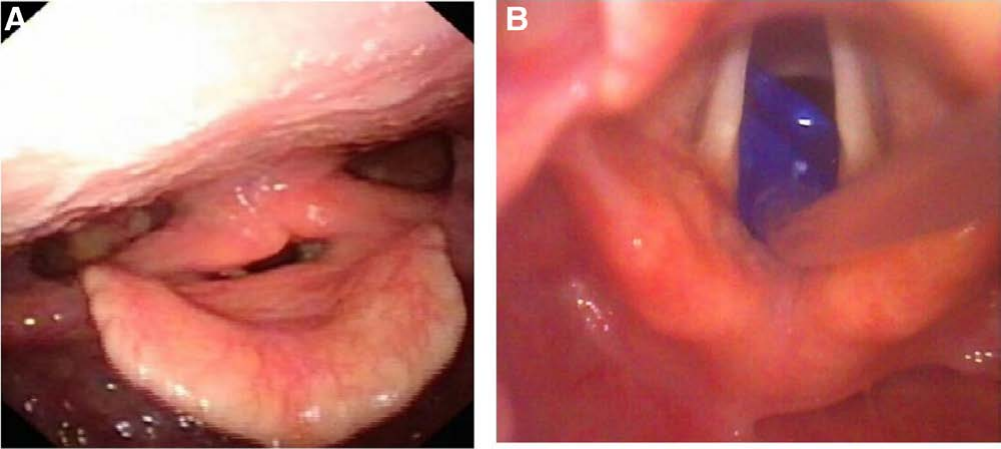
The glottic view during laryngoscopy. (A) The general view of bilateral vocal cord paralysis. (B) Insertion of bronchial blocker into the glottis.

A 50-year-old woman (weight 47.6 kg, height 157 cm) presented with a history of SLE and hoarseness, which were controlled with medication. Preoperative computed tomography revealed a lung tumor that was suspected to be malignant. She was scheduled for single-port video-assisted thoracoscopic partial lobectomy under general anesthesia. Two days before surgery, fiberoptic laryngoscopy was performed by an otolaryngologist and the result revealed bilateral vocal cord paralysis (Fig. 1A). A preoperative discussion among the anesthesiologist, rheumatologist, otolaryngologist, and thoracic surgeon was conducted and reached the following opinions: the bilateral vocal cord paralysis was likely due to SLE and the condition was stabled without requiring special treatment, moreover, DLT or endotracheal tube combined with BB might be inappropriate due to the risk of further vocal cord injury, and LMA combined with BB might be a better choice.

In the operating room, a standard anesthetic protocol was implemented, involving routine invasive arterial blood pressure monitoring, electrocardiography, pulse oximetry, end-tidal carbon dioxide, nasopharyngeal temperature, and bispectral index. Before the induction of general anesthesia, an ultrasound-guided thoracic paravertebral nerve block at T4 level was performed with 20 mL of 0.375% ropivacaine. After preoxygenation with a face mask, uneventful rapid sequence induction of general anesthesia was performed using propofol (1.5 mg/kg), sufentanil (0.4 μg/kg), and cisatracurium (0.15 mg/kg).

A well-lubricated 9-Fr BB was pre-passed through a 3-size LMA under aseptic precautions. After anesthesia induction, with the help of a video-assisted laryngoscope, the BB can be inserted through the glottis (Fig 1 B). Using the BB as a guide, we then put the LMA in place. The LMA position was confirmed by a fiberoptic bronchoscope, which showed an unobstructed view of the vocal cords and the BB was positioned in the right main bronchus. The bronchial cuff was inflated with 4 mL of air. LMA and BB were firmly fixed to each other using adhesive tape. The position of BB was reconfirmed after the patient was placed in the left lateral decubitus position. The anesthesia was maintained with propofol (2–4 mg·kg^−1^·h^−1^), remifentanil (0.05–0.15 μg·kg^−1^·h^−1^), sevoflurane (1%), and dexmedetomidine (0.5–1.0 μg·kg^−1^·h^−1^) to maintain hemodynamic stability. During OLV, the following ventilator settings were used: pressure-controlled ventilation-volume guaranteed mode; fractional inspired oxygen concentration of 0.8 to 1.0; tidal volume of 6 mL/kg; positive end-expiratory pressure of 5 cm H_2_O; I:E ratio was 1:2; and inspiratory fresh gas flow was 2 L/min. The respiratory rate was adjusted to 12 to 18 breaths/min to maintain end-tidal carbon dioxide partial pressure of 35 to 45 mm Hg.

During operative period, the lung collapse was achieved by the LMA combined with BB to provide a clear view of the operative field and the patient’s vital signs were stable. There was no audible peri-laryngeal leakage during OLV. During the whole operation, 1000 mL of fluid was infused. The operation time was 100 minutes, and the OLV time was 80 minutes. After the OLV was completed, BB was removed and tracheal suctioning was performed using a fiberscope to avoid postoperative atelectasis. Two-lung ventilation, the lung expanded normally. After the surgery was completed, the LMA was removed after the patient was fully awake without obvious hemodynamic fluctuations. Then she was transferred to postanesthesia care unit; showing a basal percutaneous arterial oxygen saturation of 100%, no pain, no sore throat, and excellent spontaneous breathing. Blood gas analysis showed no significant abnormalities. The drainage tube was removed within the next 48 hours and she was discharged home 4 days after surgery without aggravated vocal cord impairment. The final pathological examination revealed adenocarcinoma of the right inferior lobe of the lung (T1bN0M0Ia2). At the 1-month follow-up, she had fully recovered without obvious abnormalities.

## 3. Discussion

OLV, a standard technique, was used to obtain surgical conditions in thoracic anesthesia. DLTs and BBs are 2 commonly used airway devices for OLV, and each has its advantages and shortcomings. Traditionally, a DLT has been the preferred airway device for lung cancer surgery, as it could offer more rapid and better quality lung collapse.^[3]^ However, the DLTs have a curved endobronchial lumen, which might rub the vocal cords when rotating into the glottis to the target endobronchus during intubation, and patients might experience serious intubation-related laryngeal injury. It is obvious that the DLT intubation was inappropriate for the patient with bilateral vocal cord paralysis before surgery.

BBs are another airway device to achieve OLV, which need to be used combined with other airway devices. Endotracheal tubes combined with BBs are the most popular combination mode. To place and position the BBs successfully, a minimum inner diameter 8.0 mm single-lumen tracheal tube is recommended.^[4]^ In the past several years, several risk factors for intubation-related laryngeal injury were identified, including demographic factors, quality of tracheal intubation, and endotracheal tube size. However, research data showed that the incidence of postoperative hoarseness and vocal cord injury was highly correlated with the size of the endotracheal tube.^[5]^ Thus, in patients with bilateral vocal cord paralysis, a large-bore single-lumen endotracheal tube might also aggravate vocal cord impairment. In addition to single-lumen tracheal tube combined with BB, LMA combined with BB is another interesting newly described airway device for OLV. There have been successful case reports on the LMA combined with BB to achieve OLV in recent years.^[6,7]^ Different from the above reports, this case reported that LMA combined with BB was used in a patient with bilateral vocal cord paralysis. LMAs as a typical supraglottic airway device, which were not implanted in the airway, the injuries on pharyngeal and tracheal mucosa were reduced, and the incidence of postoperative complications such as pharyngalgia, edema, and hoarseness was lower.^[8]^ The BB into the desired bronchus was easy due to no crowding of tubes in the lower trachea. Thus, LMA combined with BB could be a measure for OLV where conventional lung isolation devices are more traumatic, especially for patients with vocal cord diseases.

The LMA combined with BB also has some shortcomings. Some anesthesiologists might worry that the LMA will dislodge after the patient was placed in the lateral position, which might prevent the widespread application of this method. Fortunately, second-generation LMAs are better-suited airway devices to provide higher oropharyngeal sealing pressure.^[9]^ Moreover, some remedial measures could be adopted including deepening the depth of anesthesia and repositioning LMA in an event of dislodgement.^[7]^ If the above remedial measures are still unsuccessful, a small-bore tracheal tube can be inserted via the LMA using the guidance of a fiberscope and the BB remains in situ.^[10]^

In conclusion, we succeed to use the LMA combined with BB during OLV for video-assisted thoracoscopic surgery in the patient with bilateral vocal cord paralysis, which indicated this airway device was considered a better choice when OLV is required.

## Acknowledgments

We are grateful to the patient and all the researchers, including the physicians, nurses, and technicians, who participated in this study.

## Author contributions

**Writing—original draft:** Yi Li, Yu Zhang.

**Investigation:** Yudong Zhang.

**Writing—review & editing:** Lei Meng, Chong Li, Jianli Li.
